# Individual anatomical variability and interrelations: impacts on swallowing functionality and clinical perspectives

**DOI:** 10.1590/2317-1782/e20240360en

**Published:** 2025-07-07

**Authors:** Guilherme Maia Zica, Maria Inês Rebelo Gonçalves

**Affiliations:** 1 Universidade Federal de São Paulo – Unifesp - São Paulo (SP), Brasil.

**Keywords:** Anatomy, Physiology, Biological Variation, Individual, Deglutition Disorders, Swallowing

## Abstract

Have you ever wondered during clinical practice why the manifestations of swallowing dysfunctions are so heterogeneous? For example, an individual may go through the aging process and different illnesses with functional swallowing or, in another scenario, may present different forms of dysphagia manifestation. What would be the possible factors associated with the development or not of dysphagia besides those already known? By reading a book on human anatomy, it is possible to understand the complexity of anatomical structures and their different forms and correlations. For years, there have been countless descriptions in the literature regarding the anatomical and physiological variability between individuals and how this may or may not promote changes in functionality. There are countless anatomical and physiological variations known in human beings. However, the individual and personalized approach to individual anatomical correlations of swallowing and their impact on dysfunctions, therapeutic programs and prognosis is still rarely found in the literature. In this paper, we will describe a brief history of research into individual anatomical variations in the area of health and dysphagia and the complex human evolutionary context, in an attempt to reflect on the question: would it be possible for some individuals to have an anatomy and/or physiology that is naturally more prone to swallowing dysfunctions? Mastering anatomy and physiology is fundamental for intervention in dysphagia, however, we believe that other aspects should be considered in the future for assertive and personalized assessment, planning and intervention.

When examining a human anatomy textbook, one can grasp the complexity of anatomical structures and their various forms and interrelationships. For years, numerous descriptions in the literature have addressed the anatomophysiological variability among individuals and how this may or may not lead to functional alterations. Normality encompasses the most frequent anatomical patterns; however, within this group, morphological variations related to size, sex, age, quantity, shape, and attachment of structures are acceptable, provided they do not impair function^(1,2)^. 

Anatomical and physiological variations in humans are innumerable. A substantial body of scientific research has sought to understand morphological variability and its potential impact on individuals.

In the context of physical therapy and physical education, Bonilla et al.^(3)^ propose that exercise programs should be developed based on the individual anatomical and biomechanical patterns of the musculoskeletal structures, as well as on genetic, pedagogical, and methodological aspects directly associated with the stimulus-response process, in order to reduce the incidence of injuries. In this regard, the authors advocate for the consideration of individual anatomical relationships to enhance outcomes and minimize injury risk^(3)^. 

In a systematic review, Lastoria^(4)^ aimed to describe the effect of quadriceps anatomical factors on patellar stability in humans. Although the strength of the vastus medialis obliquus has been considered a key determinant of patellar stability, the evidence remains conflicting due to the complexity inherent in analyzing this model^(4)^. 

In a systematic review conducted in 2024, Fuenzalida et al.^(5)^ described anatomical variants in the origin of the coronary arteries. The authors report that individual anatomical alterations may involve anomalous or atypical origins, as well as variations in the course, location, or shape of these vessels. Such variations are often asymptomatic and may be clinically benign. However, it is recommended that patients diagnosed incidentally and in the absence of symptoms undergo regular monitoring to prevent potential complications. Future studies may contribute to a deeper understanding of the anatomical, embryological, and physiological aspects of the numerous anatomical variants of the heart^(5)^. 

In the context of speech therapy, during clinical practice, one may question why swallowing dysfunctions present such heterogeneity. For example, an individual may age and experience various health conditions and frailty while maintaining functional swallowing, or conversely, may present with different degrees of dysphagia. What, then, are the factors contributing to the development of dysphagia, beyond the already recognized external risks?

Given the complexity of individual anatomical variations and their numerous interrelationships, it is crucial to address this aspect from the perspective of swallowing. Since its first description by Helsham^(6)^ around 1800, dysphagia has long been considered a dysfunction treated through surgical and instrumental interventions^(6)^. Since then, most publications have focused on studying dysfunctions and comparing individuals, while little is known about normal swallowing and, more importantly, its individual variations.

In 1897, Meltzer^(7)^ described swallowing as a complex neuromotor process involving the superior laryngeal nerve, based on his experimental studies with dogs and rabbits^(7)^. In his 1989 article, *Physiology of Swallowing,* published in *Dysphagia*, Dodds^(8)^ described swallowing as we know it today, detailing its structures (both soft and hard), their interrelationships, the phases (oral, pharyngeal, and esophageal), and its neuromotor control^(8)^. 

Recent studies, such as those by Wei et al.^(9)^, provide a deeper, preliminary understanding of the role of cortical and subcortical neural structures. Cortical regions are primarily responsible for initiating and coordinating swallowing upon receiving afferent input, while subcortical structures, including the basal ganglia and thalamus, regulate and control swallowing movements through the cortico-basal ganglia-thalamic-cortical circuit^(9)^. 

Despite over 200 years of scientific knowledge, the individualized approach to the anatomical relationships of swallowing and their impact on dysfunctions, therapeutic programs, and prognosis remains limited in the literature.

In 2022, Alves et al.^(10)^ conducted an in-depth study on hyoid bone displacement patterns in healthy individuals during swallowing of different consistencies. They analyzed 201 videofluoroscopic swallowing exams from 67 adults and elderly individuals without swallowing disorders. Seven displacement patterns were identified, with the horizontal pattern being the most frequent, showing variations between men and women. The individual displacement pattern remained consistent across all three consistencies (thin liquid, puree, and solid)^(10)^. In other words, although there is a basic consensus on what constitutes normal swallowing, numerous factors remain unknown, such as the individual displacement patterns of the structures.

In a 2023 meta-analysis by Hartfield et al.^(11)^, aimed at describing the current understanding of the individual anatomical factors that determine the collapsibility of the upper airways in obstructive sleep apnea, relevant findings were made regarding the variability in the anatomy and morphology of the oropharyngeal structures. The meta-analysis identified four key anatomical variables associated with airway collapsibility: hyoid position, tongue volume, pharyngeal length, and waist circumference^(11)^. These factors may also affect swallowing; however, it remains unclear whether an individual may be more susceptible to dysphagia than another due to prior physical or functional characteristics.

Randolph et al.^(12)^, in their book *Why We Get Sick: The New Science of Darwinian Medicine* (1996), raise the following questions: “Why do we need the protective mechanisms of the lower airways and the cough reflex? Wouldn't it be much safer and easier if our airways and digestive tracts were completely separated? What functional reason exists for this crossover?” Their answer is simple: none. The explanation is evolutionary, not functional. All vertebrates share the same “design flaw”: the intersection of the digestive and respiratory systems. This “anatomical and physiological flaw” is not present in other animal groups, such as insects and mollusks, which have independent respiratory and digestive systems^(12)^. 

Given the complex historical and evolutionary context of our body and its numerous variations, could some individuals have an anatomy and/or physiology that is naturally more prone to swallowing dysfunctions? Unfortunately, there is still no answer to this complex question. However, dialogue and reflection in both research and clinical practice from an individualized perspective are undoubtedly crucial.

In the future, biomechanical models that quantify the relative importance of these anatomical factors in determining the individual morphological and functional relationship may help identify deviations and the ideal intervention for each patient. Many anatomical and structural factors still require more detailed studies, including: pharyngeal spaces and their variations; hyoid position, angulation, and displacement; tongue position and volume; the shape and alignment of the jaw and maxilla; as well as the timing and sequencing of events during swallowing^(1,2,9-11,13,14)^.

A comprehensive understanding of human anatomy and physiology is crucial for the rehabilitation of patients with dysphagia; however, we assert that additional individual factors must be considered in the assessment, planning, intervention, and understanding of therapeutic limits in a personalized approach.
