# Supplementary material for: Individual anatomical variability and interrelations: impacts on swallowing functionality and clinical perspectives
Source: Codas. 2025 Jul 7;37(4):e20240360. doi: 10.1590/2317-1782/e20240360en (PMC12259064; doi:10.1590/2317-1782/e20240360en)
Supplement: Translation

# Translation: Variabilidade e relações anatômicas individuais: impactos na funcionalidade da deglutição e perspectivas clínicas

**DOI:** 10.1590/2317-1782/e20240360pt

**Authors:** Guilherme Maia Zica, Maria Inês Rebelo Gonçalves

**Affiliations:** 1 Universidade Federal de São Paulo – Unifesp - São Paulo (SP), Brasil.

**Keywords:** Anatomia, Fisiologia, Variação Biológica Individual, Disfagia, Deglutição

## Abstract

Você já se perguntou, durante a prática clínica, o porquê das manifestações das disfunções de deglutição serem tão heterogêneas? Por exemplo, um indivíduo pode envelhecer e passar por diferentes contextos de adoecimento e fragilidade com uma deglutição funcional ou, em outro cenário, pode apresentar diferentes formas de manifestação da disfagia. Para além das etiologias já conhecidas, quais seriam então os possíveis fatores associados ao desenvolvimento ou não de uma disfagia? Ao se debruçar sobre um livro de anatomia humana é possível compreender a complexidade das estruturas anatômicas e suas diferentes formas e correlações. Há anos observam-se inúmeras descrições na literatura em relação à variabilidade anatomofisiológica entre os indivíduos e como isso pode ou não promover alterações na funcionalidade. São incontáveis as variações anatômicas e fisiológicas conhecidas nos seres humanos. No entanto, a abordagem individual e personalizada sobre as relações anatômicas individuais da deglutição e seu impacto nas disfunções, programas terapêuticos e prognóstico ainda é escassa na literatura. Neste artigo vamos descrever um breve histórico das pesquisas em relação às variações anatômicas individuais na área da saúde e disfagia e o complexo contexto evolutivo do nosso corpo, na tentativa de refletir sobre o seguinte questionamento: seria possível alguns indivíduos apresentarem uma anatomia e/ou fisiologia naturalmente mais propensa a disfunções de deglutição? É evidente que a compreensão profunda da anatomia e fisiologia é fundamental para a atuação em disfagia, porém, acreditamos que outros aspectos futuramente deverão ser considerados para avaliação, planejamento e intervenção assertivas e personalizadas.

Ao se debruçar sobre um livro de anatomia humana, é possível compreender a complexidade das estruturas anatômicas e suas diferentes formas e correlações. Há anos observam-se inúmeras descrições na literatura sobre a variabilidade anatomofisiológica entre os indivíduos e como isso pode, ou não, promover alterações na funcionalidade. A normalidade contempla os padrões anatômicos mais frequentes; porém, são admissíveis, nesse grupo, variações morfológicas relacionadas ao tamanho, gênero, idade, quantidade, forma e fixação das estruturas, desde que não prejudiquem suas funções^(1,2)^. 

São incontáveis as variações anatômicas e fisiológicas conhecidas nos seres humanos. Existem inúmeros estudos científicos que buscam compreender a variabilidade morfológica e seu impacto nos indivíduos.

Na fisioterapia e na educação física, por exemplo, Bonilla e colaboradores^(3)^, em 2022, sugerem que os exercícios sejam planejados com base nos padrões anatômicos e biomecânicos das estruturas musculoesqueléticas individuais, bem como em aspectos genéticos, pedagógicos e metodológicos diretamente relacionados ao processo de estímulo-resposta, para mitigar a ocorrência de lesões. Ou seja, consideram as relações anatômicas individuais com o objetivo de alcançar melhores resultados e reduzir a chance de lesões^(3)^. 

Lastoria^(4)^, em uma revisão sistemática, buscou descrever o efeito dos fatores anatômicos do quadríceps na estabilidade patelar em seres humanos. Embora a força do vasto medial oblíquo tenha sido considerada um determinante da estabilidade patelar, há evidências conflitantes devido à complexidade da análise nesse modelo^(4)^. 

Em uma revisão sistemática realizada em 2024, Fuenzalida e colaboradores^(5)^ descrevem variantes anatômicas da origem das artérias coronárias. Os autores afirmam que as alterações anatômicas individuais podem ocorrer por origens anômalas ou diferentes, bem como alterações em seu destino, localização ou forma. Tais alterações são assintomáticas e podem ser inofensivas. Porém, recomenda-se que pacientes cujo diagnóstico tenha sido feito incidentalmente e na ausência de sintomas sejam submetidos a controles periódicos para prevenir complicações. Estudos futuros podem melhorar a compreensão anatômica, embriológica e fisiológica das inúmeras variantes anatômicas no coração^(5)^. 

Em relação à fonoaudiologia, durante sua prática clínica, você já se perguntou por que as disfunções de deglutição são tão heterogêneas? Por exemplo, um indivíduo pode envelhecer e vivenciar diferentes contextos de adoecimento e fragilidade com uma deglutição funcional ou, em contrapartida, com diferentes quadros de disfagia. Quais seriam, então, os fatores associados ao desenvolvimento de uma disfagia para além dos riscos externos já conhecidos?

Dentro desse contexto da complexidade anatômica individual e suas infinitas correlações, observamos a urgência de dialogar sobre este aspecto na perspectiva da deglutição. Desde seu primeiro relato descrito na literatura por Helsham, por volta de 1800, a disfagia apresentou-se, por anos, como uma disfunção tratada por modalidades de tratamentos cirúrgicos e instrumentais^(6)^. Desde então, a maioria das publicações é voltada ao estudo de disfunções e à comparação entre os indivíduos. Pouco se sabe sobre a deglutição considerada normal e, principalmente, suas variações individuais.

Meltzer^(7)^, em 1897, em seus estudos experimentais com cães e coelhos, já descrevia a deglutição como um processo neuromotor complexo, com a participação do nervo laríngeo superior^(7)^. Dodds^(8)^, em seu artigo intitulado “Physiology of swallowing”, publicado em 1989 na revista *Dysphagia*, já descrevia a deglutição como conhecemos hoje: todas as suas estruturas (moles e duras), as relações entre elas, suas fases (oral, faríngea e esofágica) e seu controle neuromotor^(8)^. 

Atualmente, como em estudos de Wei e colaboradores^(9)^ de 2024, já é possível encontrar uma descrição mais profunda e preliminar do papel das estruturas neurais corticais e subcorticais. Em suma, as regiões corticais são principalmente responsáveis pela iniciação e coordenação da deglutição após receber informações aferentes, enquanto as estruturas subcorticais, incluindo gânglios da base e tálamo, são responsáveis pelo controle e regulação do movimento durante a deglutição por meio do circuito córtico-gânglios da base-tálamo-cortical^(9)^. 

Apesar de um vasto conhecimento científico descrito há mais de 200 anos, a abordagem individual e personalizada sobre as relações anatômicas da deglutição e seu impacto nas disfunções, programas terapêuticos e prognóstico ainda é escassa na literatura.

Alves e colaboradores^(10)^, em 2022, realizaram um profundo estudo sobre os padrões de deslocamento do osso hióide em indivíduos saudáveis, considerando seus deslocamentos durante a deglutição de diferentes consistências. Foram analisados 201 exames de videofluoroscopia da deglutição de 67 indivíduos adultos e idosos sem distúrbios de deglutição. Os autores descrevem sete padrões de deslocamento do osso hióide, sendo o padrão mais frequente o horizontal, com variações na distribuição entre homens e mulheres. O padrão de deslocamento individual foi mantido durante a deglutição das três diferentes consistências (líquida fina, pastosa e sólida)^(10)^. Ou seja, apesar de já haver um consenso básico sobre a deglutição considerada normal, há inúmeros fatores ainda desconhecidos, como os padrões de deslocamento individuais.

Em uma revisão com meta-análise de Hartfield e colaboradores^(11)^, de 2023, com o objetivo de descrever a compreensão atual sobre os fatores anatômicos individuais que determinam a colapsibilidade das vias aéreas superiores na apneia obstrutiva do sono, os mesmos encontraram achados relevantes na compreensão da variabilidade da anatomia e morfologia dos órgãos fonoarticulatórios. A meta-análise identificou quatro variáveis anatômicas principais associadas à colapsibilidade da via aérea: a posição do hióide, volume da língua, comprimento da faringe e circunferência da cintura^(11)^. Estes fatores também podem interferir na deglutição; entretanto, ainda não sabemos se um indivíduo pode ser mais suscetível à disfagia do que outro devido a características físicas ou funcionais prévias.

Randolph et al.^(12)^, no livro *Why We Get Sick: The New Science of Darwinian Medicine* (Por que ficamos doentes: a nova ciência da medicina darwiniana), publicado em 1996, debatem os seguintes questionamentos: “Por que precisamos dos mecanismos de proteção das vias aéreas inferiores e do reflexo de tosse? Seria muito mais seguro e fácil se nossas vias aéreas e alimentares fossem completamente separadas. Qual razão funcional existe para esse cruzamento?” A resposta para eles é simples: nenhuma. A explicação é evolutiva, não funcional. Todos os vertebrados compartilham a mesma “falha de design”: a interseção do sistema digestivo e respiratório. Essa “falha anatômica e fisiológica” não se apresenta em outros grupos de animais, como insetos e moluscos, que têm os sistemas respiratório e digestivo independentes^(12)^. 

Considerando o complexo contexto histórico e evolutivo do nosso corpo e suas inúmeras variações, seria possível que alguns indivíduos apresentassem uma anatomia e/ou fisiologia naturalmente mais propensa a disfunções de deglutição? Infelizmente, ainda não há resposta para essa complexa pergunta. Porém, o diálogo e a reflexão na pesquisa e na prática clínica sobre uma perspectiva individualizada são, sem dúvida, fundamentais.

No futuro, modelos biomecânicos que quantifiquem a importância relativa desses fatores anatômicos na determinação da relação morfológica e funcional individual podem ajudar a identificar o desvio e a intervenção ideal para cada paciente. Muitos fatores anatômicos e estruturais requerem estudos mais detalhados, como: espaços faríngeos e suas variações; posição, angulação e deslocamento do hióide; posição e volume da língua; forma e ajuste de mandíbula e maxila; e o tempo e sequenciamento dos eventos durante a deglutição^(1,2,9-11,13,14)^.

É evidente que a compreensão profunda da anatomia e fisiologia humana é fundamental para a atuação em disfagia; porém, acreditamos que outros aspectos individuais deverão ser considerados para avaliação, planejamento, intervenção e compreensão do limite terapêutico de forma personalizada.
